# Patient Controlled Analgesia for Adults with Sickle Cell Disease Awaiting Admission from the Emergency Department

**DOI:** 10.1155/2016/3218186

**Published:** 2016-03-29

**Authors:** Josue Santos, Sasia Jones, Daniel Wakefield, James Grady, Biree Andemariam

**Affiliations:** ^1^New England Sickle Cell Institute, Division of Hematology-Oncology, University of Connecticut Health Center, 263 Farmington Avenue, Farmington, CT 06030, USA; ^2^Connecticut Institute for Clinical and Translational Science, 263 Farmington Avenue, Farmington, CT 06030, USA; ^3^Department of Community Medicine and Health Care, University of Connecticut Health Center, 263 Farmington Avenue, Farmington, CT 06030, USA

## Abstract

*Background.* A treatment algorithm for sickle cell disease (SCD) pain in adults presenting to a single emergency department (ED) was developed prioritizing initiation of patient controlled analgesia (PCA) for patients awaiting hospitalization.* Objectives.* Evaluate the proportion of ED visits in which PCA was started in the ED.* Methods.* A two-year retrospective chart review of consecutive SCD pain ED visits was undertaken. Data abstracted included PCA initiation, low versus high utilizer status, pain scores, bolus opioid number, treatment times, and length of hospitalization.* Results.* 258 visits resulted in hospitalization. PCA was initiated in 230 (89%) visits of which 157 (68%) were initiated in the ED. Time to PCA initiation was longer when PCA was begun after hospitalization versus in the ED (8.6 versus 4.5 hours, *p* < 0.001). ED PCA initiation was associated with fewer opioid boluses following decision to admit and less time without analgesic treatment (all *p* < 0.05). Mean pain intensity (MPI) reduction did not differ between groups. Among visits where PCA was begun in the ED, low utilizers demonstrated greater MPI reduction than high utilizers (2.8 versus 2.0, *p* = 0.04).* Conclusions.* ED PCA initiation for SCD-related pain is possible and associated with more timely analgesic delivery.

## 1. Introduction

Acute, severe pain episodes are the hallmark of sickle cell disease (SCD) and are frequently managed in the emergency department (ED). Optimal treatment of these acute pain episodes requires bolus dosing of intravenous opioids and frequent reassessments of pain, both of which are time-consuming for ED nurses and often lead to delays in care [1]. This is particularly true once patients are deemed to need hospitalization for continued analgesia as patients often wait for prolonged periods of time in the ED before a hospital bed becomes available. It is during this time of transition between providers as well as physical location that patients can experience delays in pain reassessment and analgesic delivery.

Although the American Pain Society guidelines recommend patient controlled analgesia (PCA) for the management of sickle cell pain among hospitalized patients [2], there is no consensus as to the appropriate timing of PCA initiation. Thus, patients generally do not receive PCA until after they are transferred to the hospital floor. The failure to initiate PCA in the ED is likely due to lack of recognition that PCA would be useful in this setting, lack of training regarding PCA initiation and its advantages, logistical complexities regarding storage of PCA pumps, and the need for large concentrated volumes of opioids [3].

As part of a quality improvement project to enhance care of adults presenting to our hospital's ED with SCD-related pain, a fast-track pain management algorithm was developed and implemented [4]. A key component of the algorithm was the initiation of PCA for those SCD patients who were awaiting admission to the hospital.

The objective of this study is to evaluate the proportion of ED visits in which PCA was utilized among SCD patients awaiting admission for continued treatment of an acute pain episode. Comparisons between ED visits in which PCA was begun in the ED versus following transfer to a hospital bed were made including mean pain score reduction, need for additional bolus opioid therapy following decision to admit, time from last opioid bolus to PCA start, and hospital length of stay. The effect of ED utilization frequency on reduction in mean pain intensity was also explored.

## 2. Materials and Methods

### 2.1. Study Design and Setting

A two-year retrospective chart review (January 2012 to December 2013) of all ED visits for SCD-related pain was undertaken according to the University of Connecticut institutional review board (IRB) policies. The study was deemed to be exempt from full IRB review. The setting was a single academic hospital ED that had implemented a SCD pain management algorithm two years prior to the start of the study period. A key component of the algorithm was the initiation of PCA in the ED by the ED physician for visits requiring admission for SCD-related pain.

Given that use of PCA in the ED was a new practice, significant education of ED staff took place prior to algorithm implementation. Each ED nurse was assigned an online case-based module and attended an in-person PCA pump competency education session. ED physicians and clinical pharmacists were educated on the use of the clinical algorithm, the use of high concentration parenteral opioids, and how to write the PCA orders based on patient response to bolus opioid therapy in the ED. Additionally, pharmacy administration was engaged to insure the logistics of initiating PCA in the ED were in place.

### 2.2. Methods of Measurement

Electronic medical records were used to extract demographic and clinical data for each visit. A standardized data collection tool along with clear definitions for reliable versus missing data was used to ensure consistency. Collected demographics included gender, age, and race. Clinical data included hemoglobin phenotype, frequency of ED visits over the two-year period, pain scores (Numerical Rating Scale of 0–10), total number of bolus opioids administered between the time of physician decision to admit and the start of PCA, and number of bolus opioids received on the hospital floor before PCA initiation. The following specific time points were also abstracted: ED physician's decision to admit, PCA initiation time, hospital floor arrival, hospital length of stay, and last bolus opioid administration prior to PCA initiation. Abstracted time points were used to calculate the following time frames: absolute time to PCA initiation, time between last opioid in the ED and first opioid on the floor, and time to starting PCA after hospital floor arrival.

Initial ED pain score was defined as the first pain score recorded upon registration to the ED. The final ED pain score was the last pain score recorded prior to transfer from the ED to the hospital floor. ED utilization was defined as the number of times a unique patient presented to any ED during the two-year study period independent of whether the visit resulted in a hospital admission. High ED utilizer visits were defined as those from patients who experienced 10 or more ED visits within the two-year study period. Low ED utilizer visits were defined as those from patients with less than 10 ED visits within the two-year study period. Outpatient records were reviewed to document whether patients had experienced an ED visit at a neighboring hospital during the study period. Such visits were included when determining high versus low ED utilizer status.

### 2.3. Outcome Measures

The primary outcome measure of this study was the proportion of ED visits for SCD-related pain in which a PCA was initiated by the ED physician while the patient was boarding the ED awaiting admission to the hospital. Secondary outcome measures included the following comparisons between visits in which a PCA was initiated in the ED versus following hospitalization: absolute time to PCA initiation, change in pain intensity while in the ED, need for additional bolus opioids after decision to admit, time from last bolus opioid to PCA start, and hospital length of stay. The effect of high versus low ED utilizer visit status on change in pain intensity while in ED was also measured.

### 2.4. Statistical Analysis

For all analyses, visit is the unit of analysis. For numerical outcomes approximately normally distributed, two-sample *t*-tests were used (e.g., pain scores during the visit, the within-visit differences in pain score, and the number of boluses). The time from last opioid bolus until PCA initiation and the absolute time to PCA initiation were not normally distributed and were assessed with the Wilcoxon rank-sum test. Categorical outcomes (e.g., opioid boluses after decision to admit: yes versus no) were analyzed using Pearson's chi-squared test. All data analyses were conducted using SAS 9.4 (SAS Institute, Cary, NC).

## 3. Results

As demonstrated in Table 1, there were 258 ED visits for SCD-related pain requiring admission from the ED among 52 unique patients. Fifty-six percent of the unique patients were female with a mean age of 29 years. The most common phenotype was hemoglobin SS disease. There was a median of 6 ED visits per unique patient over a two-year period. Nearly one-third of the unique patients were classified as high ED utilizers based on having at least 10 ED visits over the two-year study period. PCA was employed for pain management in 230 (89%) of these visits. Among visits during which PCA was utilized, 68% (*n* = 157) were initiated in the ED while the remainder (32%, *n* = 73) were begun after arrival on the hospital floor. There were no documented adverse events associated with ED PCA utilization including the need for opioid reversal with naloxone.

Clinical outcomes were compared between ED visits in which PCA was begun in the ED versus those visits in which PCA was initiated on the hospital floor (Table 2). The visit types were similar with respect to presenting pain intensity (NRS pain score 9.3 versus 9.1, *p* = 0.44) as well as improvement in pain intensity prior to transfer from the ED to the hospital ward (NRS mean pain score reduction 2.3 versus 2.1, *p* = 0.9). Similarly, there was no statistically significant difference in mean pain intensity reduction between cohorts when comparing ED initial pain score, ED final pain score, and hospital floor initial pain score with 4-hour and 24-hour posthospitalization pain score.

However, as demonstrated in Table 3, among those visits where PCA was initiated in the ED, low ED utilizer visits demonstrated significantly greater reduction in mean pain intensity compared to high ED utilizer visits (2.8 versus 2.0, *p* = 0.04). Among just high or low utilizers, there was no notable difference between those initiated in ED compared to the hospital (both *p* > 0.60).

As shown in Table 2, in comparison to visits in which PCA was initiated on the hospital floor, visits in which PCA began in the ED resulted in patients being less likely to receive bolus opioid therapy following the physician's decision to admit (72% versus 45%, *p* = 0.0001). Of those visits where bolus opioids were given after physician's decision to admit, those which began PCA in the ED recorded an average of 4-fold fewer boluses prior to PCA initiation (0.6 versus 2.7, *p* = 0.003). Importantly, the median absolute time to PCA initiation was nearly twice as long for visits in which PCA was begun on the hospital floor versus in the ED (8.6 hours versus 4.5 hours, *p* < 0.001). Additionally, the duration of time from last opioid bolus to start of PCA was significantly shorter among visits that received PCA in the ED (1.5 versus 3.5 hours, *p* = 0.0001). Visits in which PCA was not initiated in the ED waited a median of 1.4 hours for PCA to begin after arrival on the hospital floor. No significant difference was found in hospital length of stay between the visit types (6.9 versus 6.8 days, *p* = 0.87).

## 4. Discussion

Although similar strategies have been published in pediatrics [5, 6], to our knowledge we are the first to report the use of PCA in the ED for adults with SCD who are awaiting admission. Our data demonstrate that, following the initiation of a SCD pain management algorithm that prioritized the initiation of PCA in the ED, the majority of visits for SCD-related pain requiring admission did indeed receive PCA while boarding the ED. The absolute time to PCA initiation was twice as long for visits in which PCA was begun on the hospital floor versus in the ED. Additionally, wait time between last bolus opioid treatment and initiation of PCA was two hours shorter among visits in which PCA was initiated in the ED.

A study among pediatric SCD patients also demonstrated a reduction in time between final bolus opioid dose and initiation of PCA in the ED [5]. However, to our knowledge, we are the first to show that the initiation of PCA in the ED is associated with a reduction in the number of additional boluses of opioid analgesia given after decision to admit. Further study may elucidate whether this indeed impacts ED work flow by comparing ED nursing time needed to start PCA versus administering additional opioid boluses.

Our study did not identify an association between ED PCA administration and magnitude of change in pain score, suggesting that continued intermittent bolus opioid therapy may have similar analgesic effect as PCA-based therapy when measured using a standard numerical pain scale. However, we did demonstrate that visits among less frequent ED utilizers did, in fact, demonstrate a significant reduction in pain intensity when placed on PCA in the ED. This suggests that less frequent utilizers may be more likely to benefit from early initiation of PCA-based therapy and warrants further investigation.

Similar to the pediatric study by Melzer-Lange et al. [5], we did not detect a difference in hospitalization length of stay between the visit types. This suggests that earlier initiation of PCA may not impact duration of acute pain. However, neither of our studies controlled for other comorbidities that may have affected length of stay such as concurrent infection, development of acute chest syndrome, and psychosocial barriers to discharge.

## 5. Limitations

There are several limitations to our study that impact the generalizability of our findings to other hospitals. This was a single academic center study whose ED adopted a SCD acute pain management algorithm that prioritized the initiation of PCA while in the ED for patients awaiting hospital admission. In addition, ED physicians and nurses were trained in sickle cell pain management and in PCA use. Our study does underscore, however, that ED physicians and nurses can be trained in PCA use and that the potential logistical roadblocks around providing this treatment modality in the ED to adult SCD patients can be overcome.

Our study would be strengthened by elucidating why PCA was not initiated in the ED, a clear deviation from the algorithm. Unfortunately, this information was rarely documented in the medical chart and is reflective of a limitation in the retrospective study design. Similarly, we could not reliably abstract the amount of opioids administered via PCA as this was not recorded in the medical record in uniform fashion. Thus, we could not compare, for example, the total amount of opioid utilized between cohorts in the first few critical hours of hospitalization.

Another limitation of our study is that we did not directly measure patient or provider satisfaction. However, further investigation in this area could evaluate whether improvement in opioid administration wait time afforded by initiation of PCA in the ED has an impact on the experience of SCD patients who have been demonstrated by others to experience delays in acute pain care [7, 8]. We also acknowledge that in this observational cohort there are subjects that appear in both time periods, and application of the statistical tests that assume independence may not perform optimally. Readers should consider this when looking at the *p* values for those tests, which could be biased toward lower values.

## 6. Conclusions

We have demonstrated that it is possible to initiate PCA to adult patients with SCD-related pain boarding the ED. Although our findings suggest the clinical benefit is strongest among low utilizers, further study of this treatment modality is warranted.

## Figures and Tables

**Table 1:** ED visit characteristics.

Visits requiring admission	258
Unique patients	52
Gender (% female)	56%
Age, mean (SD)	29 (9)
Race (% black)	90%
Phenotype	71% SS^a^
# of ED visits^b^	6 [3, 13]
% high ED utilizers	31%
PCA initiated, *n* (% yes)	230 (89%)
Location of PCA initiation	
ED, *n* (%)	157 (68%)
Hospital, *n* (%)	73 (32%)

^a^SS = hemoglobin SS disease.

^b^Over a two-year period; median [interquartile range].

**Table 2:** Visit characteristics by PCA initiation location.

	PCA-ED	PCA-hospital	*p* value
	*n* = 157	*n* = 73	
Pain scores			
Initial ED pain score, mean (SD)	9.3 (1.1)	9.1 (1.1)	0.44
Final ED pain score, mean (SD)	7.0 (2.0)	7.0 (2.5)	0.68
Change in pain score in ED, mean (SD)^a^	2.3 (1.9)	2.1 (2.5)	0.90
Bolus opioids given after decision to admit			
% yes	45%	72%	0.0001
Total number received before PCA, mean (SD)	0.6 (0.8)	2.7 (1.3)	0.003
Number received on hospital floor before PCA, mean (SD)	—	1.8 (5.9)	—
Treatment time, hours			
Absolute time to PCA initiation^b^	4.5 [3.4, 5.6]	8.6 [6.1, 18.1]	<0.001
Last opioid bolus (either in ED or in hospital) to PCA initiation^b^	1.5 [0.9, 2.2]	3.5 [2.3, 5.5]	0.0001
Time to starting PCA after hospital floor arrival^b^	—	1.4 [0.7, 10.1]	—
Time between last opioid in ED and first opioid on hospital floor^b^	—	3.6 [2.4, 5.4]	—
Inpatient length of stay, days, mean (SD)	6.9 (7.7)	6.8 (5.7)	0.87

^a^Difference in mean pain intensity between initial and final ED pain scores.

^b^Median [interquartile range].

**Table 3:** Change in ED pain score^a^ by site of PCA initiation and visit utilizer type, mean (SD).

	Visit utilizer type		
	High	Low	*p* value
PCA-ED	2.0 (1.8)	2.8 (2.2)	*0.04*
PCA-hospital	1.9 (2.0)	2.5 (3.1)	*0.22*

^a^Difference in mean pain intensity between initial and final ED pain scores.

## Additional Points

A single emergency department implemented an algorithm prioritizing initiation of patient controlled analgesia for adults with sickle cell disease awaiting hospitalization. A postalgorithm chart review of 258 consecutive emergency department visits demonstrated that 68% of these visits began patient controlled analgesia in the emergency department while awaiting admission. Compared to visits where patient controlled analgesia commenced after hospitalization, initiation in the emergency department was associated with fewer opioid boluses and a two-hour improvement in time between physician decision to admit and initiation of patient controlled analgesia. Measurable analgesic benefit was confined to visits from less frequent emergency department utilizers.
